# Erysipelas

**Published:** 1859-04

**Authors:** C. C. F. Gay

**Affiliations:** One of the Consulting Surgeons to the Buffalo General Hospital


ART. IV.—*Erysipelas. Its Constitutional Origin and Treatment.* The Annual Oration delivered before the Erie County Medical Society. By C. C. F. Gay, M. D., one of the Consulting Surgeons to the Buffalo General Hospital.

*Mr. President and Gentlemen:*

In compliance with your action at the last meeting of this society, which made it incumbent on myself to deliver the annual address, I propose to offer, briefly, a few general observations upon Erysipelas, having especial reference to its constitutional origin and treatment.

My only apology for obtruding this subject upon your attention at this time is, that I have hitherto had better opportunities for observation, a better practical knowledge, and I may add, sadder experience with this, than, perhaps, with any other disease which it has been my province to treat.

The term erysipelas is employed in a very vague and indefinite sense. There is nothing in its etymology that indicates its pathology. It is used in our current medical literature, without prefix or suffix, as both the generic and specific term, to denote each and every variety of the disease, leaving us to adopt that which a detailed account of its treatment would suggest.

With us the disease so rarely occurs as to attract little notice, while in many other localities, it contributes largely to swell the aggregate amount of disease.

In order to exhibit its ratio of mortality in this city with other diseases, I have prepared a tabulated statement from the reports of the health physician for the past four years.

There were reported for

| | Females. | Males. | Total No. of Deaths. |
|---|---|---|---|
| 1854, . . | 1 | 0 | 1 |
| 1855; . . . | 3 | 5 | 8 |
| 1856, . . | 2 | 6 | 8 |
| 1857, . . | Sex not given. | | 4 |
| Total number for four years, . . | | | 21 |

Number occurring each month:

| Month. | No. of Deaths. |
|---|---|
| January, . . . . . . . | 2 |
| February, . . . . . . . . | 1 |
| March, . . . . . . . | 6 |
| April, . . . . . . . . | 5 |
| May, . . . . . . . | 1 |
| June, . . . . . . . . | 0 |
| July, . . . . . . . | 0 |
| August, . . . . . . . . | 2 |
| September, . . . . . . . | 0 |
| October, . . . . . . . . | 1 |
| November, . . . . . . . | 3 |
| December, . . . . . . . | 0 |
| Total number, . . . . | 21 |

The whole number of deaths occurring in this city from all causes during the same time, were 8880, making one death from erysipelas in every 442, or a fraction over $\frac{1}{2}$ of 1 per cent.

I may here be permitted to remark that the value of these reports is very much impaired from the fact of there being no allusion to the specific form or duration of the disease.

Ignoring its frequency and mortality, other reasons of sufficient magnitude present themselves, such as will not absolve the physician, as the guardian of the public health, from giving to it that full amount of thought and consideration which any disease merits and demands.

Could reliable statistics be obtained, (assuming the disease to be common elsewhere,) they would afford abundant and indubitable proof that the chronic affections of a large per cent. of the whole number of those who resort to the tricks of empiricism for their cure are derived from, and occur as a sequence to an acute attack of the disease under consideration. It has become a too common occurrence with the versatile and unstable, after recovering from an erysipelas, discovering perchance a red patch or blotch upon the surface, alarmed for the healthfulness of their blood, to resort to the whole round of popular nostrums, and after freely imbibing without relief, to turn away from everything legitimate in medicine, to seek relief in the infinitesimal doses of Hahnemann, or the wet sheet of hydropathy.

Waving all greater claims to our consideration, here are minor ones which should arrest the attention of every physician who cherishes in his heart the welfare of his profession.

I shall not presume to offer any observations, or describe in detail its symptoms or treatment. Endeavoring to avoid all unnecessary divisions into distinct and separate species, I shall assume simple erysipelas to be the true type of the disease; phlegmonous and malignant, to be results or modifications of it; all having a common origin and pathology.

The term traumatic, which only signifies an exciting cause, becomes insignificant when employed to indicate a distinct species. It would be equally appropriate to designate other species by the terms zymotic and telluric.

Sporadic and epidemic are significant expressions only so far as treatment is involved.

The disease has a pathology peculiar to itself, possesses a self-limitation, and assumes so many different forms and phases as justly entitles it to the appellation of a protean malady. It is subject to, and obeys the same general laws as do the other exanthemata; but unlike them, does not possess the power to protect the system against its own recurrence.

It is primarily a disease of the blood mass, to which, however, there may be exceptions. "Influences, especially of a mechanical kind, are so strictly local, that it would be far-fetched to derive all local disorders from a general casual disease. The latter would, perhaps, be a transfer of the alienation locally produced."

To attempt to prove that this was always a general disease, would threaten to mislead us into the error of exclusive humoralism. Nevertheless it may often exist in the system in a latent state, only awaiting the presence of the exciting cause fully to develop it.

The topical inflammation is but a localization of a general disease or its local manifestation. I apprehend, however, it is often regarded and treated as the primary affection.

John Higginbottom, F. R. C. S., Nottingham, on Nitrate of Silver in Erysipelas, says:

> "During a period of more than twenty years, I have attended a great number of cases of erysipelas, many of them extremely severe in their nature, and during that period I have lost only one patient, a middle aged female, in whom the erysipelas was attended with very severe *constitutional* symptoms, with sore throat," &c.

Now from this statement, it is legitimate to infer first, that he regarded the local inflammation as the primary disease; and secondly, that the only case of true erysipelas treated by him during that long period, proved fatal when the very local means were used, upon which he almost exclusively relied for its cure. These differences in regard to its pathology, must of necessity lead to discrepancies in treatment and errors of treatment. As the local inflammation in its characteristics, unchecked and untrammeled in its progress by local applications, is not unlike an inflammation induced by artificial vesication, I would suggest that as "nature is everywhere busy by the silent operation of her own forces," endeavoring to cure disease, she may have instituted this as a revulsive means of protection to the delicate structures of the brain, structures most liable to implication: else, why the choice of the face and scalp as its location in preference to any other. Do we not, as the interpreters of nature's laws, imitate this process in the treatment of other diseases?

The danger to be apprehended is not so much because of the intensity of the local inflammation without, as from the fire within; the two conditions being homologous, the one being derived from and dependent upon the other.

*General Treatment.* Here great contrariety of opinion exists among medical men: while one class are advocates of the tonic plan, another places most reliance upon the opposite.

A few years since, a distinguished physician of Philadelphia recommended venesection for an ordinary case in private practice. At the same time and place, I saw at the hospital stimulants administered and pushed as nearly to the point of intoxication as could be induced.

When such palpable differences exist; when the medical light and experience of yesterday is snuffed out and extinguished by the physician of today, who becomes competent to decide?

Having read some years since a paper written by Dr. Corliss, which was published in the Transactions of the State Society, in advocacy of the success attendant upon the use of quinine and wine, I at once adopted his suggestions and prescribed tonics with an indiscrimination that "knew no variableness, neither shadow of turning," but soon became convinced that what was indicated for malignant, was not so good for the simple form of disease.

A case came under my observation occurring in the person of one whose predilections were strongly in favor of hydropathy, the sum total of whose treatment consisted of two blue pills and one dose of castor oil. For another, whom I was called to attend, who was apparently much debilitated, I prescribed quinine and wine, which evidently served as a tonic for the disease, if not for the patient. Here I conceive were errors of omission and of commission; while the one took too little medication, the other took too much. The one dated his complete convalescence two months after the attack, when copious supuration from a dozen boils gave exit to the poison within; the other, hers, some weeks afterward, to the effects of a mercurial purge. The tonic plan, though not always admissible, with the profession, I believe, greatly preponderates. It is almost always judicious in the extremes of life, and in constitutions impaired by excesses. It is, perhaps, always a disease of debility; indeed the meagre statistics I have exhibited go to show it to be a disease of the spring months, when the constitution has become impaired by the rigors of a winter. In an ordinary case, however, I cannot believe tonics called for, or that depletion is admissible in any case.

The best general plan of treatment, in my esteem, may be summed up and embraced in the almost obsolete but nevertheless appropriate term, depuration. It would be an act of supererogation to enter into detail and to specify particular remedies for the full development of this plan, yet I cannot forbear the mention of one drug, which in efficiency is paramount to all others. I allude to colchicum. If there be any one drug, whose *modus operandi* is eminently that of depuration, it is this. It kindly excites all the emunctories by which the blood poison is eliminated.

When given in combination with Dover's powder, it is rendered more efficient and becomes deprived of the unpleasant effects usually attributable to it. I was led to its use for the reason that I believed there existed some points of analogy, pathologically, between it and that other form of disease for which colchicum is so much prescribed and held in so good repute.

*Local Treatment* should always be subsidiary to the general. To circumscribe the local inflammation when it attempts to spread itself over too great a surface, should be accomplished, if possible; but to attempt by the same means utterly to suppress it, I deem to be hazardous in the extreme.

Nitrate of silver has become so closely identified as a local application with this affection, that I cannot forbear to give you the words of the late Dr. Drake in relation to it. He says:

"I am compelled by experience and inquiry, to believe that the criteria by which to select the topical applications in this acute, not less many chronic affections of the skin, are as yet but little understood, and perhaps will never be very obvious. It has long seemed to me that the practice with these agents was essentially empirical and tentative; it will, I fear, continue so. That they have all done good, cannot be doubted; yet as they are always used in connection with constitutional treatment, it must be quite impossible to decide on the relative value of the external and internal. That the latter is on the whole much greater than the former, is, I think, quite certain, and it seems exceedingly probable that in many cases the cure depends entirely upon the internal, while the external applications may receive the credit. In this way we may account, in part at least, for the opposite reports made by different physicians on the efficacy of the same applications. One has used it in connection with an appropriate — the other an inappropriate internal treatment, and consequently the apparent results were different. In the midst of this uncertainty, we may presume that when the constitution of the patient is vigorous, and the phlogistic diathesis is strongly developed, the mucilaginous, farinaceous, and oleaginous applications may be most proper; while in feeble constitutions, with an early failure of the vital forces and a tendency to œdema or gangrene, the more stimulating should be chosen."

*Iodine.* The application of iodine will prove effectual in circumscribing a local inflammation when nitrate of silver fails, and is doubtless more reliable than any other agent.

*Oiled Silk.* The very best application, except when it is desirable to stimulate the surface, I have found to be the oiled silk. By its use, the surface is well protected from the atmosphere, it is light and cool, soothing and grateful to the patient. Probably no better application could be made in an ordinary case; conjoined with the iodine, it is applicable to all cases.

That form of erysipelas which exists when the local inflammation dips deep into the areola tissue, assuming the modification denominated phlegmonous, I have come to regard in many cases as the most desirable result, by virtue of the better convalescence of the patient.

As its treatment comes more immediately within the province of surgery, I dismiss it with the single remark, that tonics and stimulants are only moderately tolerated until after free exit is given to the pus.

Should there be an insufficient amount of vigor in the patient for the formation of pus, or to eliminate the blood poison from the system, or from whatever cause, should the disease become malignant, we have a very grave malady to combat. I must confess to some obscurity in regard to the applicability and significance of the term. Used in contra-distinction to benign, it always implies an intractable disorder.

A single remark or two in addition to what has already been said of the pathology of erysipelas may not be altogether inappropriate here. This modification of true erysipelas almost always involves pyæmia: especially is this true when prevalent in the hospitals. So close is the analogy between the symptoms of this affection and primitive pyæmia, that the diagnosis is involved in doubt. To know the true causes which produce death is of the first importance to the physician in treating any disease. In this disease is the brain attacked with inflammation by metastasis? Do we have phlebitis or secondary pyæmia? Questions which I leave for others more capable than myself to answer. However, it is the more rational to presume that we have in the majority of cases the latter complication; indeed, autopsies almost invariably reveal purulent depots within the joint cavities. It is, perhaps, so far as treatment is concerned, of no practical utility to know when the one condition or the other exists. Should incipient pyæmia be present, the disease is amenable to treatment; beyond this our art ceases to have control, and the patient must sooner or later succumb.

The indications of treatment are evidently to prevent these purulent depôts, and to ward off the secondary results of inflammation. To accomplish which, tonics and stimulants are demanded from the first, and should be administered with boldness, for they are beyond a doubt our chief reliance.

Much importance has hitherto been placed upon the use of the muriated tincture of iron. But I have had an experience with it so far short of success, that with myself, Fowler's solution and quinine have long since taken precedence of it.

An *erysipelatous inflammation* holds so close a relation with the disease, I have considered as to merit brief notice in this connection.

It may occur independently of, but more frequently, after an acute attack of simple erysipelas. It is vexatious both to the physician and patient. There are few maladies which give more trouble or less satisfaction to the general practitioner. It almost deserves the distinctive title of *relapsing erysipelas*. It clearly indicates the presence of the erysipelatous diathesis and although the simple form may have had its rise, progress, and decline, still the specific influence has not yet become extinct. This condition may result from an untimely suppression of the local inflammation of a general disease, or a transfer of it. It is in any event a sequence of blood lesion and constitutional disturbance. Indeed a local inflammation of this character can so seldom exist, *per se*, independently of some lesion of the general system, that to argue in favor of its constitutional origin would seem superfluous.

Rokitansky says:

"When, owing to whatever cause, a local disease has been checked in its development, it subsides only to reäppear in another part, often with augmented force and with the supervention of a new general disease." * *

Suppuration is equivalent to the extinction of the discrasis; without this issue, it is liable to localize itself again in other organs, or the same tissue between which there is intimate relation of sympathy.

The first thing to be done toward its treatment is to "unlearn the Willanean nosology and diagnosis," to look beneath the surface and to study the nature and causes of that vitiated condition of the blood, which, if it does not originate the disease, renders it at least proof against local treatment and ordinary remedies.

"By rectifying whatsoever is obviously wrong in the general system, we put the patient into a condition in which the local disease has a chance of getting well; and sometimes this is all we have to do; the *vis medicatrix naturæ* will accomplish the rest."

The best remedy, conjoined with an appropriate constitutional treatment which suggests itself, though seemingly a severe one, is the seton.

Having myself had recourse to it in two or three instances, in the treatment of this affection, I can vouch for its efficacy in doing more towards eradicating this peculiar discrasis and the restoration of the patient to health in one week, than can be accomplished in a much longer period of time by any other therapeutical means.

By thus effecting a speedy and permanent cure; empiricism, whether appearing in the form of flesh and blood or of pills and potions, becomes deprived of large and bountiful supplies of aliment upon which it has hitherto fed like the cormorant, and fattened, because of the credulity of those who are its most willing dupes.

